# The Rules of Four: A Systematic Approach to Diagnosing Common Musculoskeletal Conditions of the Knee

**DOI:** 10.51894/001c.11765

**Published:** 2020-01-30

**Authors:** Shawn Lerew, Steven Stoker, Shivajee Nallamothu

**Affiliations:** 1 Orthopedic Surgery McLaren Oakland Hospital

**Keywords:** musculoskeletal, physical exam, knee

## Abstract

Musculoskeletal symptoms are consistently one of the most commonly cited reasons for visits to ambulatory care centers every year, with knee pain accounting for approximately one-third of the reported complaints. Previous studies have demonstrated that many non-orthopedic physicians report a lack of confidence in performing clinical musculoskeletal knee examinations. “The Rules of Four” approach presented in this paper is designed to present a systematic and concise method to musculoskeletal examination of the knee within a memorable format. The approach allows for the timely diagnosis of common musculoskeletal injuries while aiding in directing further treatment and diagnostic testing. This method will ideally allow medical students and non-orthopedic physicians alike to confidently and effectively evaluate patients with complaints of knee pain in ambulatory care settings.

## INTRODUCTION

Musculoskeletal symptoms are consistently one of the most commonly cited reasons for visits to primary care providers in their offices or in ambulatory care centers every year. Of these primary care visits, knee pain accounts for approximately one-third of chief complaints.^1,2^ In 2016, 10.6 million visits were performed in the United States for knee symptoms which was just slightly less than for hypertension (11.4 million) and more than for throat symptoms (9.7 million), fever (8.8 million), and diabetes mellitus (8.5 million).^1^

Previous studies have demonstrated that many non-orthopedic physicians report a lack of confidence in performing clinical musculoskeletal examination.^3^ As many as 78 - 82% of new medical school graduates also fail to demonstrate basic competency in musculoskeletal examination as defined by both internal medicine and orthopedic program directors.^4^ These studies demonstrate the need for a systematic approach to musculoskeletal examination that can be easily taught and applied by both orthopedic and non-orthopedic physicians alike.

The “Rules of Four” approach described in this paper is a memorable and effective systematic method for a knee examination developed by third author Dr. Nallamothu to help non-orthopedic physicians confidently arrive at diagnosis when evaluating a patient’s knee complaint. This method allows the physician to confidently arrive at a working diagnosis and make decisions concerning the next step to take.

With this approach, the knee is divided into three columns each with four corresponding examination points and a 4^th^ “column” comprised of the four main ligaments of the knee. The examination points correspond to palpable landmarks that aid in the diagnosis of common musculoskeletal complaints. The four columns combined with the four points in each column is why this systematic approach was named the “Rules of Four”. In this article, we will describe the application of this approach in the musculoskeletal examination of the knee.

The knee should be evaluated in a thorough but efficient manner. After some experience, the knee exam can lead the physician to very quickly and accurately arrive at an initial working diagnosis. The first step is to obtain a complete history of the knee complaint. Next, an exam of the knee including inspection and palpation is performed. These two very important aspects along with the history may already direct the examiner towards a working diagnosis.

An inspection of the patient and knee will provide details on patient body habitus, gait, deformity, spinal issues, etc. Palpation can also provide information on effusion (i.e., intra-articular pathology), warmth (to identify possible infection), pain tolerance, etc. The method described in this paper is an organized sequence to evaluate a patient’s knee to provide a more directed diagnosis for the primary care physician in their initial evaluation. This may lead the primary care physician to recommend further treatments that may involve therapy, injections, bracing, rest, activity modification, or direct further imaging (e.g., an MRI) before referring the patient to an orthopedic surgeon.

In the following sections, we will divide the knee exam into three columns: medial, middle, and lateral, with each column having four corresponding examination points. Finally, we will discuss the 4^th^ column which includes examination of the four primary ligaments of the knee: the ACL, PCL, MCL, and LCL.

NOTE: All Knee images from the Complete Anatomy application by 3D4Medical.^5^

**Figure:**
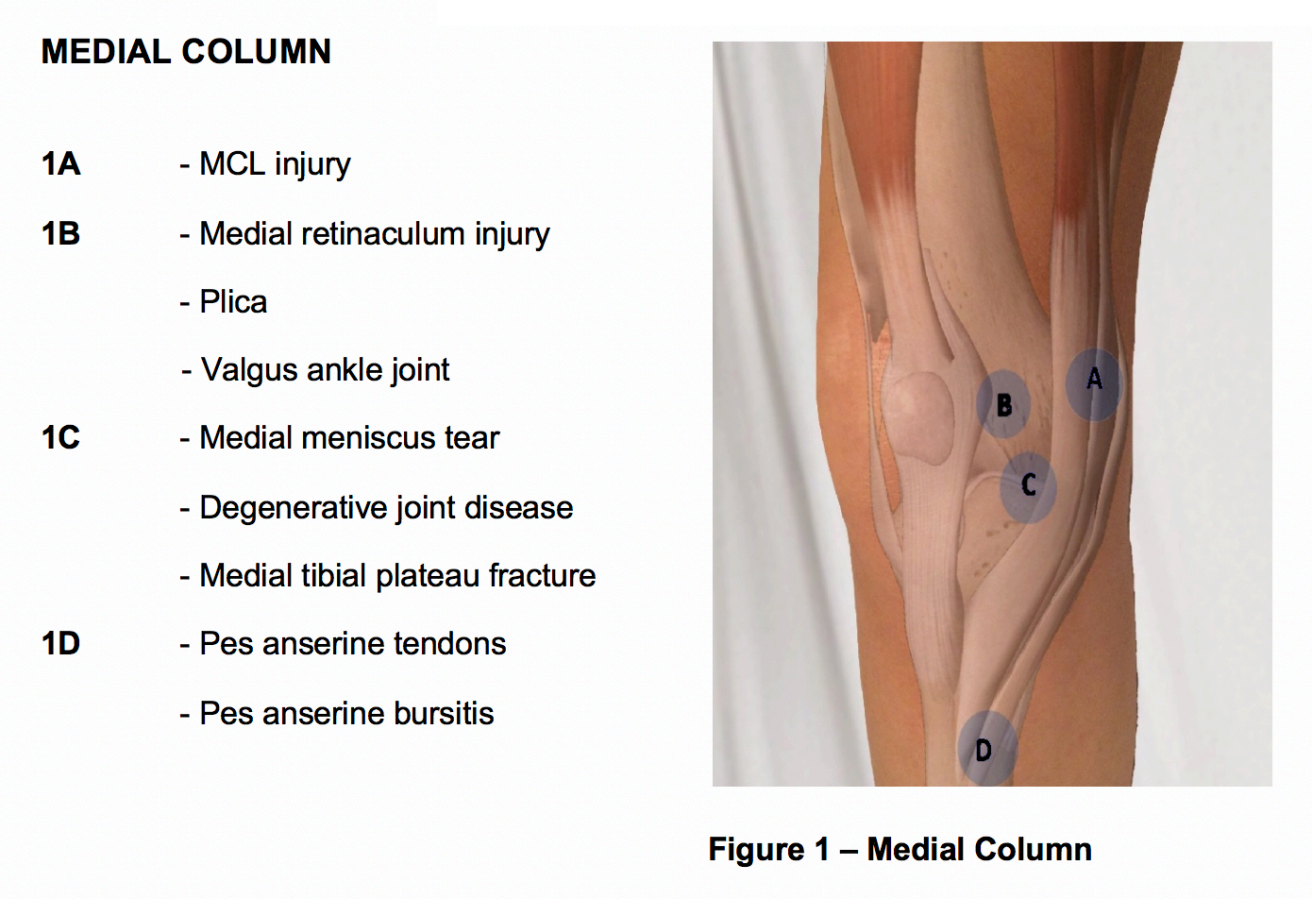
Figure 1 – Medial Column

**Figure:**
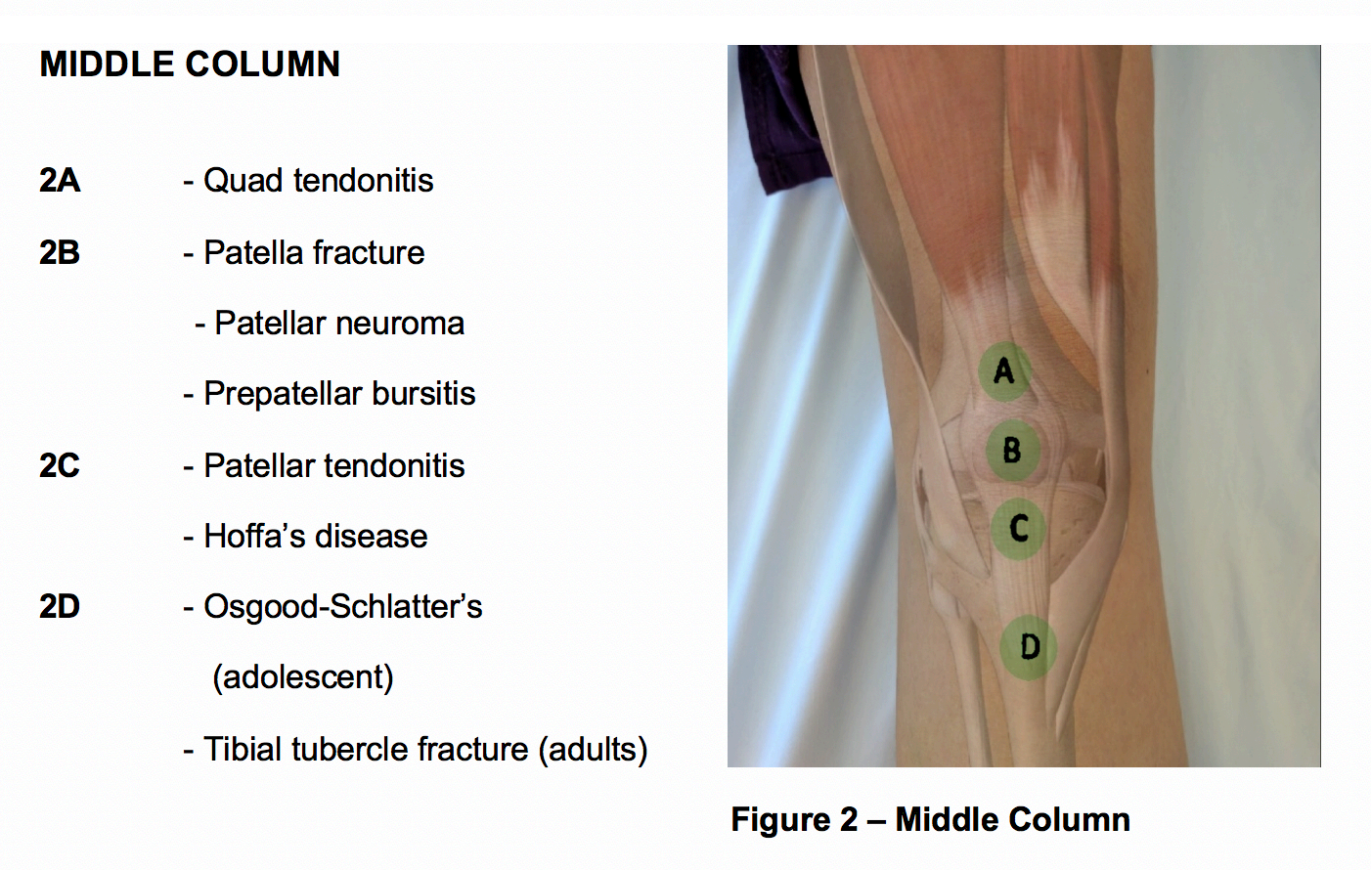
Figure 2 – Middle Column

**Figure:**
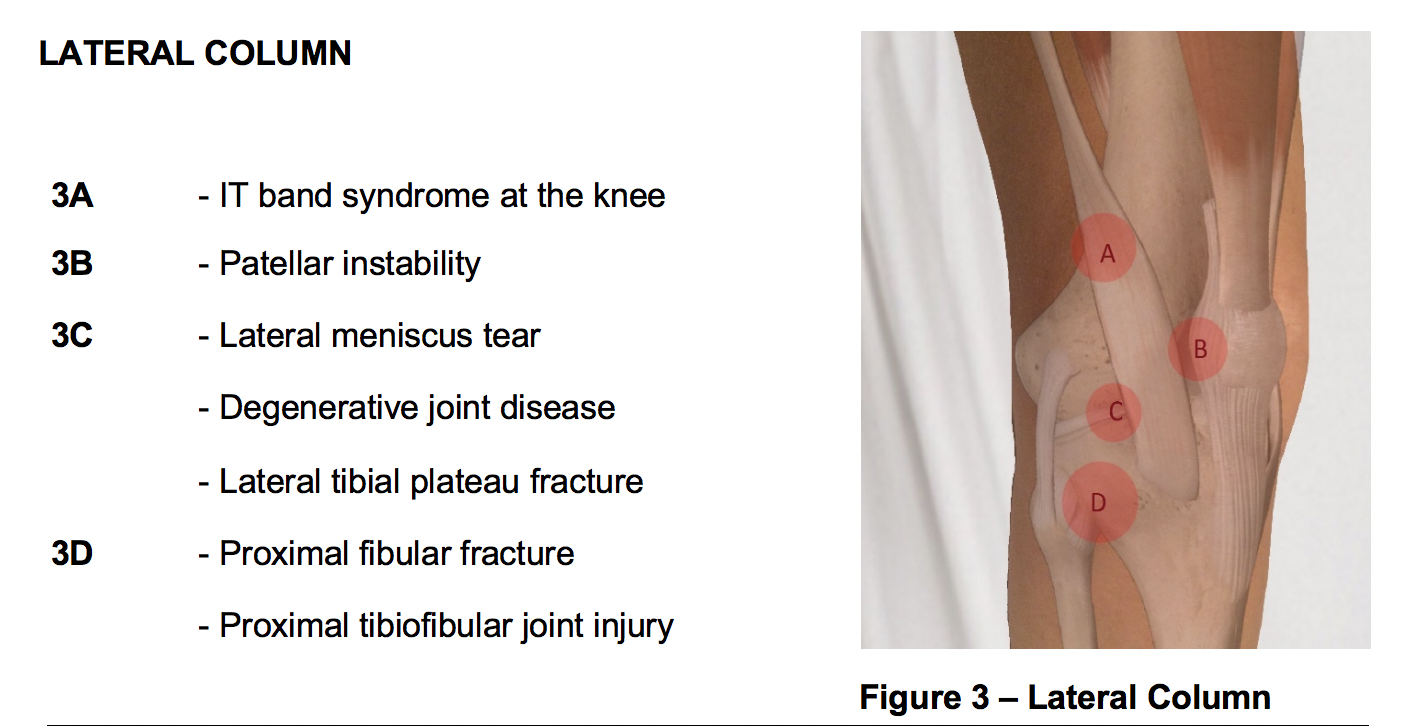
Figure 3 – Lateral Column

**Figure:**
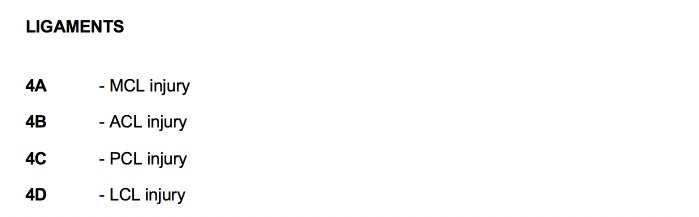
Figure 4 – Ligaments

### MEDIAL COLUMN

Common causes of knee pain that originate from the medial column of the knee are as follows: Medial collateral ligament (MCL) tear, medial meniscal tear, medial patellofemoral ligament / retinaculum (MPFL) tear, and pes anserine bursitis. If there is a recent history of significant knee trauma, a medial tibial plateau fracture should also be considered, but this is a far less common cause of medial knee pain. Utilizing the “Rules of Four” the examiner palpates the points that correspond to each diagnosis with reproduction of the patient’s pain representing a positive test.

**Figure:**
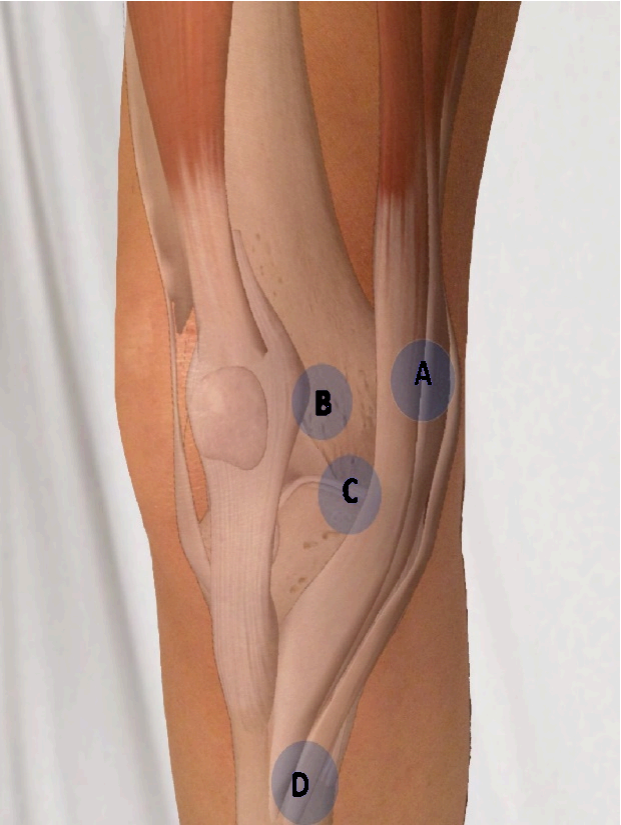
Figure 1 – Medial Column

The **first point** (“A” in figure 1) to be palpated is the medial femoral condyle, as this is the origin of the medial collateral ligament. A tear or avulsion of the MCL may result in tenderness to palpation over the medial femoral condyle. MCL injuries typically occur following a valgus-external rotation injury to the knee. The MCL is typically ruptured at the femoral insertion. A positive test should be supported by a valgus-stress test which will be discussed later.

The **second point** (“B” in figure 1) of palpation is over the medial patellofemoral ligament (MPFL) or retinaculum, which may be torn in patients with patellar instability as the patella dislocates laterally. The MPFL attaches to the superomedial border of the patella and a point between the medial femoral condyle and adductor tubercle. Tenderness to palpation over the MPFL is considered a positive test and is suggestive of a MPFL tear when associated with patellar instability. Additionally, patients with a valgus ankle joint may exhibit pain in this region due to altered biomechanics at the knee joint. A hypertrophied synovium or plica may also be palpated and is known to generate a pain response in this region.

The **third point** (“C” in figure 1) of palpation is the medial joint line. Tenderness to palpation over the medial joint line often signifies two common etiologies: medial meniscal tear and degenerative joint disease. This point is found by first palpating the inferior pole of the patella with the knee in 90 degrees of flexion and then moving medial along the joint line. Tenderness to palpation may be observed in patients with a medial meniscal tear.

It is important to palpate the along the joint line to its most posterior aspect as many tears involve the posterior horn of the medial meniscus. If pain is not elucidated but the clinician maintains a high index of suspicion for a medial meniscal tear, the tibia is then externally rotated and the knee is brought into extension while continuing to palpate the medial joint line for a painful and palpable click (i.e., Medial McMurray Test). Radiographs possibly followed by MRI can be used to confirm the diagnoses of degenerative joint disease and meniscal tear respectively.

The **fourth and final point** (“D” in figure 1) of palpation on the medial aspect of knee is the pes anserine bursa. The bursa may be visibly swollen, or the clinician may locate it by palpating slightly inferior and two finger breaths medial to the tibial tubercle. Tenderness to palpation is considered a positive finding. Hamstring muscle tightness, obesity, and valgus malalignment are often implicated as risk factors for developing pes anserine bursitis. Hamstring tightness and may be assessed by measuring the popliteal angle with the patient lying supine and comparing it to the contralateral side. Valgus malalignment may be noted clinically with inspection of the overall alignment of the knee or radiographically with full length weight-bearing X-Rays of the associated limb.

### MIDDLE COLUMN

**Figure:**
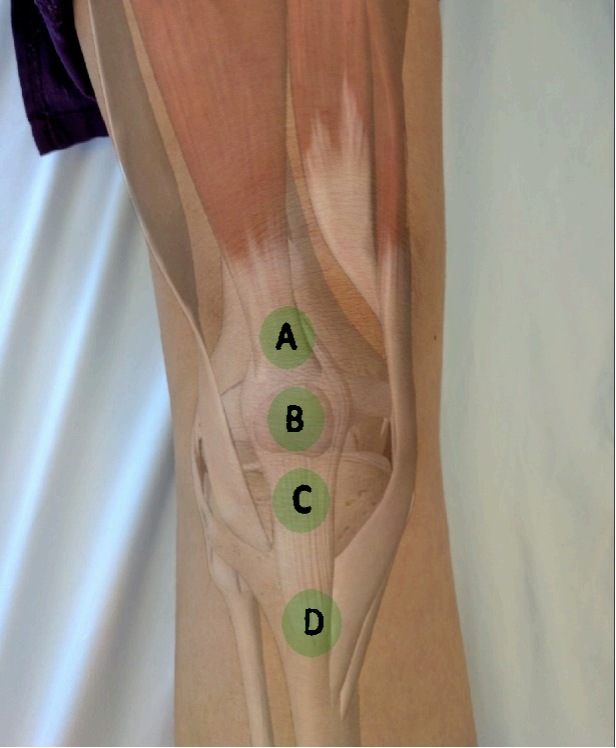
Figure 2 – Middle Column

Common causes of knee pain that originate from the middle column of the knee are as follows: quadriceps tendon rupture, quadriceps tendinitis, patellar fracture, prepatellar bursitis, patellofemoral pain syndrome, patellar tendinitis, Sinding-Larsen-Johansson syndrome, and Osgood-Schlatter disease.

The **first point** (“A” in figure 2) of palpation is the quadriceps insertion onto the superior pole of the patella. Tenderness to palpation may indicate quadriceps tendonitis. Occasionally a palpable defect may be noted indicative of a quadriceps tendon rupture. If a quadriceps tendon rupture is suspected, it is important to assess the integrity of the knee extensor mechanism by having the patient perform a straight leg raise; inability to perform a straight leg raise indicates loss of the extensor mechanism likely requiring surgical intervention.

The **second point** (“B” in figure 2) of palpation is the patella. Tenderness to palpation as well as swelling can indicate patellar fracture or prepatellar bursitis. Patellar neuroma formation following surgery is another common cause of pain in this region. The neuroma forms as a result of transection of the infrapatellar branch of the saphenous nerve. Patellar ballottement may also be assessed at this time and is an indication of significant joint effusion.

The **third point** (“C” in figure 3) of palpation is the patellar tendon insertion on the inferior pole of the patella. Palpation is best performed with the leg extended and the foot supported. The patient should be instructed to relax their leg as much as possible. The inferior pole of the patella may be further exposed by exerting pressure anterior to posterior on the superior pole of the patella. The inferior pole of the patella is then palpated; tenderness suggests patellar tendinitis (Jumper’s Knee) or patellar apophysitis (Sinding-Larsen-Johansson Syndrome) in skeletally immature athletes.

Patellar tendinitis is exceptionally common in jumping and running athletes – hence the colloquial term Jumper’s Knee. Additionally, the examiner should palpate deep to the patellar tendon both medially and laterally. Tenderness to palpation on either side of the patellar tendon, but not at its insertion is suggestive of Hoffa’s fat pad syndrome. Hoffa’s fat pad – infrapatellar fat pad – is highly vascular and innervated. Edema of the fat pad can be a source of significant pain for patients.

The **fourth point** (“D” in figure 2) to examine is the tibial tuberosity. Tenderness to palpation may indicate a tibial tubercle avulsion fracture or a traction apophysitis (i.e., Osgood Schlatter’s Disease) – both of which are commonly seen in adolescent athletes.

### LATERAL COLUMN

**Figure:**
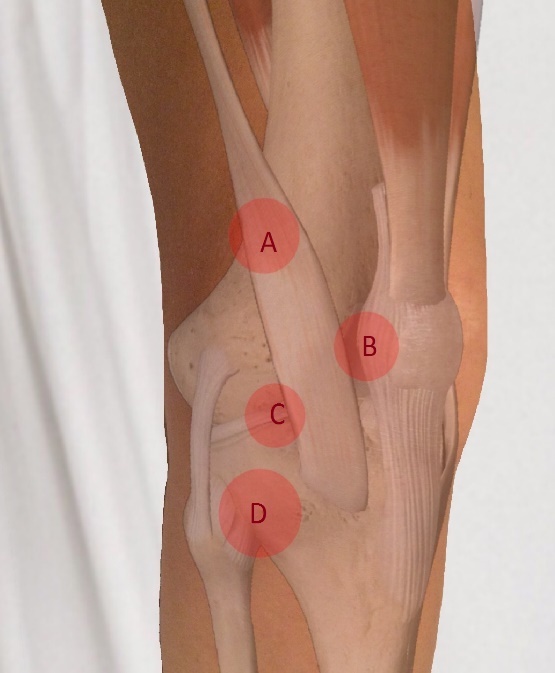
Figure 3 – Lateral Column

Common causes of knee pain that originate from the lateral column of the knee are as follows: iliotibial band syndrome (IT band syndrome), patellar instability, lateral meniscal tear, degenerative joint disease, proximal fibula fracture (i.e., Maisonneuve fracture), and proximal tibiofibular dislocation. A history of recent significant knee trauma could also be suggestive of a lateral tibial plateau fracture, but as with medial joint line pain, this is a much less common cause of lateral knee pain.

The **first point** (“A” in figure 3) to examine corresponds with IT-band syndrome, which is most commonly seen in runners and cyclists. The IT band originates on the iliac crest and inserts on the lateral aspect of the proximal tibia. It courses over the lateral femoral epicondyle prior to inserting on the proximal tibia at Gerdy’s tubercle. Tenderness to palpation at the point where the IT band courses over the lateral femoral epicondyle is suggestive of IT band syndrome.

Additionally, if the examiner has a high index of suspicion in the presence of a negative exam, the patient can be asked to lie on their contralateral side. The knee is then ranged from extension to flexion while maintaining a moderate compressive force at the lateral femoral epicondyle. Reproduction of the patient’s symptoms, particularly at 30 degrees of flexion is considered a positive test for IT band syndrome (Noble’s test).

The **second point** (“B” in figure 3) of examination is the patellar apprehension test for patellar instability. The patient is positioned in 30 degrees of knee flexion and instructed to relax their leg. The examiner then exerts a force on the medial aspect of the patella translating the patella laterally. Signs of physical discomfort or the patient attempting to straighten their leg are considered a positive apprehension test. Additionally, the examiner should note the amount of lateral patellar translation. Lateral translation of greater than 50% of the patellar width is considered abnormal and suggests patellar instability. In the setting of acute patellar dislocation, tenderness to palpation over the medial patellofemoral ligament may be elicited in addition to a positive apprehension test.

The **third point** (“C” in figure 3) of palpation is the lateral joint line. Tenderness to palpation over the lateral joint line, as is also the case for the medial joint line, signifies two likely etiologies: lateral meniscal tear and degenerative joint disease. This point is found by first palpating the inferior pole of the patella with the knee in 90 degrees of flexion and then moving lateral along the joint line. Tenderness to palpation may be observed in patients with a lateral meniscal tear.

If pain is not elucidated, but the clinician maintains a high index of suspicion for a lateral meniscal tear, the tibia is then internally rotated and the knee is brought into extension while continuing to palpate the lateral joint line for a painful and palpable click (i.e., Lateral McMurray Test). Radiographs, possibly followed by MRI, can be used to confirm the diagnoses of degenerative joint disease and meniscal tear respectively.

The **fourth point** (“D” in figure 3) of palpation is the proximal fibula. Tenderness to palpation can be elicited in patients with proximal fibula fractures or, rarely, tibiofibular dislocations. Radiographs should be obtained as the next step in evaluation.

## LIGAMENTOUS STABILITY

Finally, the four primary ligaments of the knee are assessed for stability. These include the anterior cruciate ligament (ACL), posterior cruciate ligament (PCL), medial collateral ligament (MCL), and the lateral collateral ligament (LCL). Accurate diagnosis of ligamentous injuries always involves comparison of the uninjured contralateral side.

First, the examiner assesses the integrity of the ACL. While there are several tests that can be used to evaluate the ACL (Lachman test, anterior drawer, pivot shift) research supports the Lachman test as the most valid test for ACL rupture in both acute and chronic injuries.^6^ The pivot shift test, while highly specific, has a low sensitivity when performed on acute ACL injuries. Similarly, the anterior drawer test has been shown to be neither sensitive nor specific in detection of acute ACL injuries: sensitivity 49%, specificity 58%.^6^

This may be due to difficulty in attaining enough degree of knee flexion to perform the test due to pain and hemarthrosis (condition occurring due to bleeding in the joint cavity). Additionally, hamstring muscle contracture may occur as a protective response to pain, providing a force that opposes anterior translation of the tibia during an anterior drawer test. The starting position of the Lachman test is both less painful for the patient and reduces the effect of protective muscle contraction: allowing for a more accurate test.

The Lachman test is performed with the knee in 30 degrees of flexion. The examiner then grasps and stabilizes the distal femur with one hand while translating the proximal tibia anteriorly with the other hand. An intact ACL functions to limit anterior translation and therefore will provide a solid endpoint to anterior translation. Increased anterior translation compared with the uninjured knee, or a soft endpoint, are highly suggestive of an ACL injury.

Next the integrity of the PCL is assessed by the “posterior drawer test.” The posterior drawer test is performed with the knee in 90 degrees of flexion. A posterior force is then applied to the proximal tibia and the amount of posterior translation quantified. In a normal knee the tibia will remain anterior to the femoral condyles during a posterior drawer test.

If the tibia is translated so that it is flush with the femoral condyles the PCL is likely ruptured. If the tibia is translated posterior to the femoral condyles the PCL is likely ruptured in combination with other capsuloligamentous structures. If the tibia remains anterior to the femoral condyles, but translates further than the contralateral side, a partial tear may be suspected.

The third ligament to be evaluated is the MCL. The MCL is evaluated by performing a valgus stress test with the knee in 30 degrees of flexion to isolate the ligament. The examiner should pay particular attention to the presence of a firm endpoint and the amount of medial gapping produced compared to the contralateral knee. No firm endpoint or the presence of increased medial gapping is indicative of MCL injury.

Finally, the LCL is evaluated by performing a varus stress test. The knee is flexed 30 in order to isolate the ligament. The examiner should pay particularly close attention to the presence of a firm endpoint and the amount of lateral gapping produced compared to the contralateral knee. No firm endpoint or the presence of increased medial gapping is indicative of LCL injury.

## DISCUSSION

The Rules of Four method proposed in this paper is a systematic approach designed to help non-orthopedic physicians to evaluate a patient’s knee complaint. It provides a memorable, systematic and efficient approach to mastering the knee exam. The method allows for the timely diagnosis of common musculoskeletal injuries while aiding in directing further treatment and diagnostic testing.

As discussed, many studies have pointed of the deficiencies in medical education to provide students with knowledge and confidence to evaluate a musculoskeletal complaint. Knee complaints are seen almost as commonly as hypertension and more commonly than other common complaints of fever, throat complaints, and diabetes mellitus.^1^ The Rules of Four approach will help provide non-orthopedic physicians an effective tool evaluating the 10.5 million knee complaint visits each year.

### Conflict of Interest

The authors declare no conflict of interest.
